# Immunomodulatory Treatment Strategies Targeting B Cells for Heart Failure

**DOI:** 10.3389/fphar.2022.854592

**Published:** 2022-03-08

**Authors:** Xinxin Zhang, Yuxi Sun, Ning Wang, Yanli Zhang, Yunlong Xia, Ying Liu

**Affiliations:** Heart Failure and Structural Cardiology Division, First Affiliated Hospital of Dalian Medical University, Dalian, China

**Keywords:** cardio-oncology, CD20, b cells, rituximab, cardiovascular diseases, heart failure

## Abstract

Cardio-oncology, a nascent specialty, has evolved as a concerted strategy to address the cardiovascular complications of cancer therapies. On the other hand, emerging evidence has shown that some anti-tumor drugs, such as CD20-targeted rotuximab, also have markedly cardioprotective effects in addition to treating cancers. Rituximab is a CD20-targeted monoclonal antibody and kill tumor B-cells through antibody-mediated and antibody-independent pathways, indicating that B cells participate and promote the progression of cardiovascular diseases. In this review, we mainly present the evidence that B cells contribute to the development of hypertrophy, inflammation, and maladaptive tissue remodeling, with the aim of proposing novel immunomodulatory therapeutic strategies targeting B cells and their products for the treatment of heart failure.

## Introduction

With the rapid development of early detective tools and therapeutic strategies for cancer patients, mortality rates attributed to cancer have remarkably declined. However, this improved survival status is achieved at the expense of anti-tumor treatment-induced cardiovascular diseases (CVDs), either caused by direct cardiotoxicity of anticancer treatment or preexisting CVDs or cardiovascular risk factors (Zamorano et al., 2016). Manifestations of adverse cardiovascular complications for cancer therapy include cancer therapeutics-related cardiac dysfunction (CTRCD) (reduction in left ventricular ejection fraction or heart failure), premature coronary artery disease, valvular disorders, pericardial injury, and arrhythmia. As the survival rates for patients with malignant tumors significantly increase, CVDs have become the second leading cause of long-term morbidity and mortality among cancer survivors (Howlader et al., 2010; Siegel et al., 2012; Bodai and Tuso 2015; Curigliano et al., 2016). Therefore, Cardio-oncology, a novel medical discipline focusing on the identification, prevention, and treatment of cardiovascular complications related to cancer therapy, has emerged. Currently, research on how to avoid adverse cardiovascular response and reverse the unfavorable CVD outcomes in patients with anti-tumor therapies has become a hot spot for both oncologists and cardiologists. Intriguingly, some recent studies have demonstrated that some anti-tumor drugs (such as CD20-targeted rituximab), in addition to treating cancers, also show markedly cardioprotective effects, but the specific mechanism has not been clarified (Uchida et al., 2004; Cang et al., 2012; Waldman et al., 2020). Therefore, exploring the potential value of anti-tumor drugs in the treatment of heart failure (HF) may provide new targets and ideas for remedying HF.

Increasing evidence shows that besides direct cardiotoxicity of anticancer treatment, malignant tumors are associated with CVDs through common risk factors, such as smoking, obesity, diabetes, hypertension and hyperlipidemia, diet, alcohol, exercise, age, sex, and race. The famous CANTOS study illuminated that in patients with previous myocardial infarction and a high-sensitivity C-reactive protein level of 2 mg or more per liter, anti-inflammatory therapy targeting the interleukin-1β innate immunity pathway with canakinumab not only led to a significantly lower rate of recurrent cardiovascular events, but also markedly reduced incident lung cancer and lung cancer mortality, suggesting that immune-inflammatory mechanism may be a common pathophysiological basis for cancers and CVDs (Ridker, et al., 2017a; Ridker, et al., 2017b).

## Immune Inflammatory Mechanism in Myocardial Remodeling

Inflammation, as a defensive response to various pathogens, is regulated by innate and adaptive immune systems. The innate immune system is a highly conserved system that operates by nonspecific mechanisms and functions as a first line of defense, mainly relying on macrophages, dendritic cells, neutrophils, circulating monocytes, granulocytes, and even some non-immune cells that adopt immunological functions as needed. In contrast, the adaptive immune system is highly specific and able to remember and effectively mount responses against previously encountered immunological threats, which relies on the ability of T and B lymphocytes.

In addition to resisting the invasion of pathogens, immune responses also participate in various CVDs. Adequate evidence elucidates that multiple types of immune cells are involved in the process of myocardial remodeling and HF, such as neutrophils, monocytes/macrophages, and lymphocytes. Neutrophils are the most abundant white blood cells circulating in the human peripheral blood, and they respond quickly to acute injuries. In ischemic cardiomyopathy, neutrophils infiltrate the infarcted myocardium and mediate tissue damage. Removal of neutrophils significantly attenuate myocardial necrosis caused by ischemia-reperfusion (Frangogiannis, 2014). Recent studies have shown that neutralization of protein S100A9, secreted by neutrophils, ameliorated Ang II-induced hypertension, ventricular hypertrophy and fibrosis, indicating that neutrophils could act as an important regulator in pathophysiological ventricular remodeling (Wu et al., 2014). In addition, some previous studies have shown that bone marrow-derived monocytes were recruited into myocardial tissue after Ang II infusion and differentiated into M1 type macrophages, which secreted various inflammatory cytokines and chemokines, promoting myocardial cells apoptosis and fibroblasts transdifferentiation (Wang et al., 2014). Wenzel et al. observed selective ablation of lysozyme M-positive (LysM) myelomonocytic cells by low-dose diphtheria toxin attenuated Ang II-induced blood pressure increase, improved vascular endothelial and smooth muscle dysfunction, reduced vascular superoxide formation and the expression of NADPH oxidase subunits gp91phox and p67phox in mice. Moreover, our previous studies found that angiotensin II (Ang II) activated AT1RPKA-proteasome pathway, which promotes degradation of IκBα and MKP-1 and activation of STAT1 and NF-κB, thereby leading to Th1 differentiation. Th1 cells are generally considered to be associated with the occurrence of various adverse cardiovascular diseases (Qin et al., 2018). In another research, Gröschel et al. reported mice lacking functional recombination activation gene 2 (Rag2), without clymphocytes, were protected during the transition from hypertrophy to heart failure following transverse aortic constriction (TAC) (Groschel et al., 2018). Currently, novel evidence has come to light that B lymphocytes may also play an essential role in the initiation and progression of HF through direct (by secreting antibodies) or indirect (by cytokines/chemokines secretion) pathways.

## B Cells Biology Characteristics

### B Cell Subgroups

B cells can be divided into two lineages, B1 cells and B2 cells. These 2 cell subsets differ considerably in ontogeny, function, location, and surface marker expression (Naradikian et al., 2016). In general, B1 cells (based on the expression of surface marker CD5, B1 cells can be further subdivided into B1a and B1b subsets) represent the vast majority of B cells in newborns, and mainly originate from fetal liver and omentum. In adults, B1 cells mainly exist in the pleural cavity and peritoneal cavity, and respond to T cell-independent antigens by secreting polyreactive natural immunoglobulin M (IgM) antibodies (Douna and Kuiper, 2016). B2 cells are produced postnatally, and represent the vast majority of B cells in adults, including a dominant population of follicular B cells and a smaller population of marginal zone (MGZ) B cells (Montecino-Rodriguez and Dorshkind, 2012). In a physiological state, B2 cells are produced in waves that parallel the waves of development of hematopoietic stem cells (HSCs), and circulate in lymphatic organs such as blood, thymus, spleen and lymph nodes. Functionally, B2 cells contribute to T-cell dependent humoral and adaptive immune responses through isotype class switching and affinity maturation (Montecino-Rodriguez and Dorshkind, 2012). In short, B1a cells don’t regenerate after birth but are kept alive by self-replication, while B1b cells and B2 cells are produced throughout life (Melchers, 2015).

### B Cell Surface Markers

B cells have a variety of surface molecules, which mediate different biological functions. The B cell receptor (BCR) is generally regarded as the characteristic surface marker and one of the most important receptors for B cells to sense external environment. Its most important function is as a receptor that recognizes various extracellular antigens in response to bacterial and viral infections for conferring host defense. The CD19/CD21/CD81 complex is the B-cell co-receptors, which can strengthen B cells activation signaling. CD20 is a transmembrane phospholipid protein that appears in the stages from pre-B cells to mature B cells, and is encoded by the MS4A1 gene. Notably, in addition to being expressed in normal B cells, CD20 is also expressed in B lymphocyte-derived lymphomas, leukemias, and neoplasms involved in immune and inflammatory diseases.

### B Cell Biological Function

Although B cells are traditionally known for their ability to produce antibodies, they can also perform other critical functions in the context of immune responses. B1 cells can generate natural IgM antibodies in the absence of specific stimulation, while B2 cells differentiate into plasma cells that produce high concentration of antibodies and long-lived memory B cells when the BCR is engaged by an antigen (Savage and Baumgarth, 2015). In addition, B cells could identify antigens through the BCR, internalize them for processing and present them to CD4^+^ T cells in the germinal centers. Moreover, B cells could secrete cytokines and chemokines, which exert a series of important biological effects. For example, B cells activate innate immune response through the secretion of IFN-γ, IL-6, and IL-17 (Shen and Fillatreau, 2015); promote CD4^+^ T cell polarization through both MHC-II-dependent and MHC-II-independent mechanisms; modulate the maturation and growth of lymphoid structures through secreting LTα1β2 (Shen and Fillatreau, 2015); regulate the mobilization of monocytes through production of CCL7(Zouggari et al., 2013); recruit T cell to inflamed tissue through generating PEPITEM(Chimen et al., 2015). In contrast, some B cell subsets, mainly referred to as Bregs or B10 cells, also act as negative regulators of immune response through the secretion of IL-10, IL-35, and TFG-β. These immunosuppressive B cells could support immunologic tolerance, resolve acute inflammatory response, and maintain the homeostasis of certain types of natural killer cells (Shen and Fillatreau, 2015).

## The Role of B Cells in Cardiac Remodeling and Heart Failure

### B Cells in the Naive Myocardium and Human Heart

In the naive murine heart, B cells have been reported to be the most prevalent leukocytes (Adamo et al., 2018; Bonner et al., 2012). Myocardial B cells account for almost 10% of circulating B cells which adhere to the naive heart vascular endothelium and arrest their transit when they pass through the heart. Prior studies reported that the vast majority (>95%) of myocardial B cells remain intravascular, whereas few (<5%) cross the endothelium into myocardial tissue (Adamo et al., 2020a). Ramos et al. further observed that there were two subgroups of murine B220^+^ (CD45R) lymphocytes in the naive cardiac muscle: a larger population of IgM^high^ IgD^low^ cells and a smaller population of IgM^low^ IgD^high^ cells (Ramos et al., 2017). Currently, there have been limited evidence to support the presence of B cells in normal human hearts. Data from histological analysis of autopsies indicate that B cells are present at a frequency similar to that of CD4^+^and CD8^+^ T cells in normal human hearts (Noutsias et al., 2002). Moreover, the presence of B cells in the human heart is also supported by some rare case reports of primary cardiac B cell lymphoma (Grantomo et al., 2018; Thiagaraj et al., 2018; Li et al., 2019). In human specimens, B cells have also been discovered within the pericardial fat, with a relative increase in the size of pericardial-fat-associated lymphoid clusters in patients with coronary artery disease (Horckmans et al., 2018).

### B Cells and Cardiovascular Diseases

In recent years, different mouse models have been used to explore the association between myocardial B cell and CVDs, especially in these fields of ischemia reperfusion (I/R) and myocardial infarction (MI). In a I/R mouse model, Yan and his colleagues observed that the frequency of myocardial CD19^+^ B cells markedly increased after MI, peaking between day 5 and 7 after injury and returning to baseline soon. In addition, they found that the time course of B cell numbers in the myocardium after I/R injury differed from that observed after permanent coronary ligation, with B cells peaking in number 2 days earlier and returning to baseline faster (Yan et al., 2013). Zouggari et al. studied a mouse model of permanent coronary ligation and found that B220^+^ B cells infiltrated into the myocardium and localized around the infarct zone after injury (Zouggari et al., 2013). They also explored the link between B lymphocytes and adverse left ventricular (LV) remodeling for the first time. In another fundamental study about cardiac hypertrophy and remodeling, TAC surgery was reported to induce an increase in the number of myocardial CD19^+^ B cells at 4 weeks postoperatively (Wu et al., 2017). These findings suggest that specific subsets of cardiac B cells are model-dependent, showing different function in various models.

Currently, few studies have been performed on the role of B cells in the human heart. In a study about human acute ischemic cardiomyopathy, patients with ST-segment MI had a slight decrease in the number of circulating B220^+^ cells at 1.5 h after myocardial reperfusion, which was followed by an remarkable increase above pre-reperfusion levels at 24 h after reperfusion (Boag et al., 2015). A cohort analysis of 56 patients with dilated cardiomyopathy (DCM) showed that HF patients had higher percentages of CD19^+^ B cells and actively replicating CD19^+^ B cell in peripheral blood than healthy volunteers. Additionally, they found that patients with DCM also had an increase in the percentage of TNF-producing B cells, which correlated directly with indices of cardiac dysfunction and myocardial fibrosis, whereas no change in the percentage of IL-10-producing B cells compared to healthy controls (Yu et al., 2013). In another study enrolled patients with non-ischemic cardiomyopathy, the investigators discovered that patients with HF showed an increased percentage of circulating Bregs (CD19^+^ CD5^+^ CD1d^+^ IL-10^+^ cells) in peripheral blood (Guo et al., 2015). In an *in vitro* study, mononuclear cells were isolated from patients with DCM and cultured for 48 h. The results showed that DCM patients had a lower prevalence of IL-10-producing B cells than healthy volunteers, and reduced IL-10 production efficiency was always associated with cardiac dysfunction (Jiao et al., 2018). Overall, the limited evidence provided by human studies about the involvement of B cells in cardiovascular disease suggests that the findings in animal models may be applicable to humans (Adamo et al., 2020b).

## Potential Mechanisms of B Cell Participation in CVDs

**FIGURE 1:**
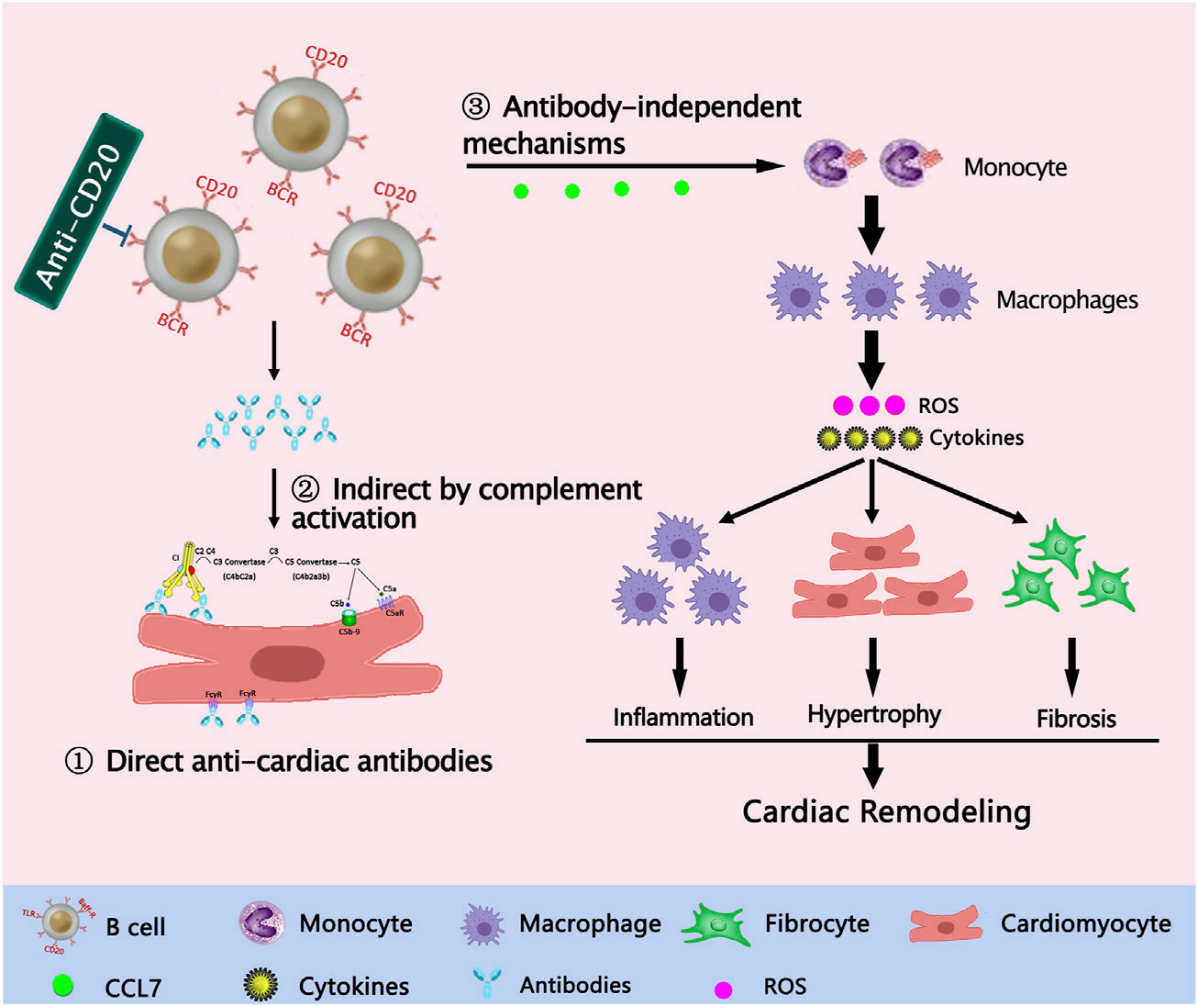
Potential mechanisms of B cell participation in cardiac remodeling and heart failure. ①Direct effects of anti-cardiac antibodies; ②Indirect by complement activation; ③ Antibody-independent mechanisms.

Emerging evidence indicates that B cells play a critical role in the context of myocardial adaptation to injury through various mechanisms, including antibody-mediated mechanisms and antibody-independent mechanisms. (Figure 1).

### Antibody-Mediated Mechanisms Contributing to Cardiac Dysfunction and Heart Failure

Antibody-dependent mechanisms contributing to cardiac injury mainly include direct effects of anti-cardiac antibodies and the activation of the complement system following the formation of antigen–antibody complexes.

#### Direct Anti-Cardiac Antibodies

A recent fundamental study has observed the important role of natural IgM antibody in myocardial I/R injury and identified a specific clone of B1 cells which could generate a type of natural antibodies that promote myocardial damage (Zhang et al., 2004; Zhang et al., 2006). Another study about myocardial I/R injury also showed that coronary artery ligation produced myocardial infarction (MI) and depressed ejection fraction, while these adverse effects were markedly reduced in Ig-deficient mice, indicating that antibodies play an important role in the development and progression of cardiac remodeling and heart failure (Keppner et al., 2018). In animal model, antibodies against specific cardiac proteins have also been shown to be sufficient to lead to cardiomyopathy (Matsui et al., 1997). For example, antibodies against troponin I were found to produce severe dilated cardiomyopathy in PD-1-deficient mice (Okazaki et al., 2003). Another cohort study identified anti-desmoglein-2 (Anti-DSG2) antibodies as a sensitive and specific biomarker for arrhythmogenic right ventricular cardiomyopathy (ARVC). In humans, the level of anti-DSG2 antibodies correlated with the burden of premature ventricular contractions; *In vitro*, the antibodies caused gap junction dysfunction, which was a common feature of ARVC. Anti-DSG2 antibodies likely explain the cardiac inflammation frequently identified in ARVC and may represent a new therapeutic target (Chatterjee et al., 2018).

A histological analysis of human myocardial tissue with end-stage HF showed immunoglobulin G (IgG) deposition was found in up to 70% of heart tissue samples (Youker et al., 2014). Almost 50% of biopsies were IgG3-positive, with a smaller proportion of C3c deposition stained positive. The presence of IgG3 and C3c in the myocardium has been proven to correlate with the duration and severity of HF(van den Hoogen et al., 2019). Moreover, both left ventricular diastolic dysfunction and end-stage HFrEF patients showed elevated levels of circulating IgG1 and IgG3, suggesting there was an antibody-mediated immune response in cardiac remodeling (van den Hoogen et al., 2019).

#### The Effects of Complement on Myocardial Injury

The complement system is an important component of innate immune responses, and can be activated through three pathways during HF. Classical pathway is initially activated by IgM or IgG antigen/antibody complexes, and induces the formation of the Membrane Attack Complex (MAC) through a series of cascade reactions (Nesargikar et al., 2012). Alternative pathway means that the complement system is activated by direct binding of bacteria and yeast independent of antibody interaction (Nesargikar et al., 2012). Lectin pathway is the most recently discovered one that is capable of activating the complement system. The initiating molecules for this pathway are collectins (mannose-binding lectin and ficolin), which are multimeric lectin complexes (Nesargikar et al., 2012).

In recent years, there has been sufficient evidence to demonstrate that the complement system participates in the development of HF. In murine sepsis models, C5a, an important protein molecule of the complement system, has been verified to play an essential role in inotropic dysfunction *via* C5aR-mediated signaling pathway (Niederbichler et al., 2006). In a murine model of hypertension, blocking C5a receptors with inhibitors mitigated cardiac hypertrophy and perivascular fibrosis, and remarkably improved cardiac function, indicating that C5a was deeply involved in adverse cardiac remodeling (Zhang et al., 2014). Moreover, C5a is an effective chemokine that attracts a variety of bone marrow derived inflammatory cells to sites of injury and activates pro-fibrotic process through modulating TGF-β/Smad2/3 signaling pathway in the heart (Nesargikar et al., 2012; Zhang et al., 2014). In addition to C5a, C5b-9, the MAC complex, has also been shown to be closely associated with HF. C5b-9 has been proved to induce tumour necrosis factor-α (TNF-α) expression in cardiomyocytes, which contributes to cardiomyocyte hypertrophy, fibrosis, and apoptosis (Zwaka et al., 2002). All of these pathophysiological processes are critical components of injury in HF(Torre-Amione et al., 1996; Yu et al., 2013). Additionally, in a cohort study, compared with healthy volunteers, patients with HF exhibited increased circulating levels of complement activated cleavage end product C5b-9, which were tightly associated with disease severity (Wang and Cai, 2020).

### Antibody-Independent Mechanisms Leading to Cardiac Injuries

In addition to acting through antibodies and complement, activated B cell could secrete cytokines and chemokines to directly modulate cardiac function and induce cardiac remodeling. B cells have been shown to be directly involved in cardiac remodeling through up-regulating cytokines of TGF-β and IL-6, and be responsible for maintaining a harmful inflammatory environment through production of TNF-α, IL-1β and IL-6 (Garcia-Rivas et al., 2020). TNF-α-secreting B cells in DCM patients were associated with increased cardiac fibrosis, as confirmed by late gadolinium enhancement on cardiac magnetic resonance imaging and elevated serum type iii pro-collagen levels (Yu et al., 2013).

B cells also exert biological effects by altering cardiac function through the secretion of chemokines. A recent study reported that mature B lymphocytes selectively produce chemokines CCL7, which can induce Ly6C^hi^ monocyte mobilization and recruitment to the heart, leading to worsened tissue injury and deterioration of myocardial function in a mouse model of ischemia-reperfusion injury (Zouggari et al., 2013). In this study, genetic (Baff receptor deficiency) or antibody-mediated (CD20-or Baff-specific antibody) depletion of mature B cells impeded CCL7 production and monocyte mobilization, reduced cardiac injury and improved heart function. The authors further found that the circulating concentrations of CCL7 and BAFF in patients with acute myocardial infarction were markedly higher compared to healthy controls, and elevated levels of CCL7 and BAFF always predicted an increased risk of death or recurrence of myocardial infarction (Zouggari et al., 2013). In another study on acute kidney injury, the researchers also found that B cells produced chemokine CCL7, with the potential to facilitate neutrophil and monocyte recruitment to the injured kidney, aggravating renal impairment. CCL7 blockade in mice markedly reduced myeloid cell infiltration into the kidney and ameliorated acute kidney injury (Inaba et al., 2020).

## Anti-CD20 Mediated B Cell Depletion as a Therapeutic Strategy for Heart Failure

In cancer patients, the capacity of immune system to detect and eliminate malignant cells is dramatically impaired. Immunotherapy aims at retraining the affected immune system and restoring their anti-cancer functions (Lobenwein et al., 2021). In recent years, a large number of studies have shown that compared with traditional chemotherapy approaches, immunotherapy drugs have shown significant effects in improving overall survival, and have gradually become an important pillar of systemic treatment for various cancer types (Waldmann 2003; Pardoll 2012). As an important immunotherapy drug, monoclonal antibodies aim to disrupt the essential activities of tumor cells and cancer immune evasion in order to increase apoptosis and immune recognition of cancer cells. The new drug could inhibit tumor growth and metastasis by targeting specific receptors that are crucial for signaling pathways in dysregulated cancer cells and immune cells (Waldman et al., 2020). CD20 is an atypical tetraspanin expressed only on mature B cells and becomes a clinical target for the treatment of B-cell lymphoma. Rituximab is a CD20-targeted monoclonal antibody that binds to CD20 molecules and kills tumor B-cells through antibody-dependent cytotoxicity (ADCC) and complement-dependent cytotoxicity (CDC). Mechanistically, rituximab crosslinks the CD20 receptor present on most B cells, leading to FcγR-mediated B cell depletion (Uchida et al., 2004). FcγRs are present on cardiac fibroblasts and cardiomyocytes, and FcγR signal transduction promotes cardiomyocyte fibrosis by activating the apoptotic pathway, reduces calcium transients and cell shortening, and induces cardiomyocyte apoptosis (Staudt et al., 2007; Haudek et al., 2008; Keppner et al., 2018).

In clinical practice, CD20 depleting agents are not only approved for B cell-related cancers, but also increasingly used on- and off-label for autoimmune diseases, such as rheumatoid arthritis (RA), multiple sclerosis (MS) and systemic lupus erythematosus (SLE) (Cang et al., 2012). Clear evidence that B cell depletion may be effective for autoimmune therapy comes from an MS study which showed rituximab treatment can increase remission rates and reduce the development of new lesions (Hauser et al., 2008). Orrelizumab, another CD20-specific cytolytic antibody, has also been shown to be suitable for the treatment of patients with relapsed or primary progressive forms of MS(Boz et al., 2019; Sabatino et al., 2019; Fox et al., 2021). In addition, studies in mouse models of Type 1 Diabetes Mellitus (T1DM), MS, and RA have also shown the protective effect of B cell depletion, which is consistent with an increasing number of highly suggestive studies (Serreze et al., 1998; Yanaba et al., 2007; Matsushita et al., 2008). In short, CD20 targeted monoclonal antibodies provide excellent efficacy in the treatment of both B cell derived tumors and autoimmune diseases.

In addition to anti-tumor and anti-autoimmune diseases, anti-CD20 treatments have been found to exert beneficial effects on cardiac remodeling and HF. Using a TAC pressure overload model of myocardial hypertrophy, Ma et al. found administration of rituximab markedly improved heart function, and suppressed heart chamber dilation, myocyte hypertrophy, fibrosis and oxidative stress through regulating calcineurin A, ERK1/2, STAT3, TGFβ/Smad2/3 and IKKα/β/NF-κB signaling pathways, suggesting that rituximab may be a promising drug for treating hypertrophic disease (Ma et al., 2019). In a mouse model of ischemia-reperfusion injury, researchers observed that the mice treated with anti-CD20 monoclonal antibodies had reduced cardiac adverse LV remodeling and improved cardiac function following permanent coronary artery ligation of the left anterior descending artery (Zouggari et al., 2013). Moreover, Tschope et al. presented a case series of six patients with subacute and chronic endomyocardial biopsy-confirmed CD20^+^ B-lymphocyte-associated DCM treated with standard HF therapy in combination with rituximab. Of these, five DCM patients improved clinically several weeks after receiving a standard infusion protocol with rituximab, as indicated by decreased NYHA functional class, improved left ventricular ejection fraction, and reduced B-cell infiltration in endomyocardial biopsies (Sanchez-Trujillo et al., 2019). Based on these encouraging data, small-scale clinical trials investigating the effect of B cell depletion in the context of heart failure with reduced ejection fraction are being planned (ClinicalTrials.gov Identifier: NCT03332888) (44), and clinical trials in patients with ST-segment myocardial infarction are ongoing (ClinicalTrials.govmIdentifier: NCT03072199) (Sanchez-Trujillo et al., 2019).

There is also some literature supporting other B cell-targeting therapies. In a basic study, using a pressure overload mouse model induced by Ang II-infusion, the researchers found that administration of monoclonal antibodies targeting CD22, another important molecule present on the surface of B cells, led to decreased myocyte hypertrophy and myocardial fibrosis, as well as reduced concentrations of inflammatory cytokines, such as IL-1β and TNF-α(Cordero-Reyes et al., 2016). Belimumab, BAFF-specific antibody, specifically targets the BAFF receptor and acts as an effective B cell depleting agent. Currently, Belimumab is mainly used for the treatment of resistant SLE in adults, showing excellent efficacy in reducing flares and overall disease activity when combined with standard therapy (Blair and Duggan, 2018). Additionally, the most established agents targeting BCR signaling are Bruton tyrosine kinase (BTK) inhibitors and PI3K isoform-specific inhibitors, and their introduction into the clinic is rapidly changing the treatment of B-cell malignancies (Burger, 2019). In the future, BTK inhibitors and PI3K isoform-specific inhibitors could provide another therapeutic possibility for H, although there may be some controversy.

## Conclusion

While most attention has been paid to the cardiovascular toxicity associated with anti-tumor therapy, some drugs have also shown great cardioprotective effects. Anti-CD20 agents have been preliminarily proved to improve left ventricle function in patients with refractory DCM, suggesting that B cells play a prominent role in cardiac remodeling and HF. Mechanically, B cells promote cardiac injuries through antibody-dependent and antibody-independent pathways. Notably, the specific B-cell subpopulation mediated myocardial damage needs to be further verified in the future research, as this would support trials of more specific therapeutics. Overall, it would be of great significance to identify the excellent cardioprotective effects of certain anti-tumor medications and apply them to the treatment of vascular diseases.

## References

[B1] Adamo L. Rocha-Resende C. Mann D. L. (2020b). The Emerging Role of B Lymphocytes in Cardiovascular Disease. Annu. Rev. Immunol. 38, 99–121. 10.1146/annurev-immunol-042617-053104 32340574

[B2] Adamo L. Rocha-Resende C. Lin C. Y. Evans S. Williams J. Dun H. (2020a). "Myocardial B Cells Are a Subset of Circulating Lymphocytes with Delayed Transit through the heart.". JCI Insight 5. 10.1172/jci.insight.134700 PMC7098796 31945014

[B3] Adamo L. Staloch L. J. Rocha-Resende C. Matkovich S. J. Jiang W. Bajpai G. (2018). "Modulation of Subsets of Cardiac B Lymphocytes Improves Cardiac Function after Acute injury.". JCI Insight 3. 10.1172/jci.insight.120137 PMC6124442 29875326

[B7] Blair H. A. Duggan S. T. (2018). Belimumab: A Review in Systemic Lupus Erythematosus. Drugs 78 (3), 355–366. 10.1007/s40265-018-0872-z 29396833

[B8] Boag S. E. Das R. Shmeleva E. V. Bagnall A. Egred M. Howard N. (2015). T Lymphocytes and Fractalkine Contribute to Myocardial Ischemia/reperfusion Injury in Patients. J. Clin. Invest. 125 (8), 3063–3076. 10.1172/JCI80055 26168217 PMC4563749

[B9] Bodai B. I. Tuso P. (2015). Breast Cancer Survivorship: a Comprehensive Review of Long-Term Medical Issues and Lifestyle Recommendations. Perm J. 19 (2), 48–79. 10.7812/TPP/14-241 PMC4403581 25902343

[B10] Bönner F. Borg N. Burghoff S. Schrader J. (2012). Resident Cardiac Immune Cells and Expression of the Ectonucleotidase Enzymes CD39 and CD73 after Ischemic Injury. PLoS One 7 (4), e34730. 10.1371/journal.pone.0034730 22514659 PMC3326036

[B11] Boz C. Terzi M. Özer B. Turkoglu R. Karabudak R. Efendi H. (2019). Comparative Analysis of Fingolimod versus Teriflunomide in Relapsing-Remitting Multiple Sclerosis. Mult. Scler. Relat. Disord. 36, 101376. 10.1016/j.msard.2019.101376 31473488

[B12] Burger J. A. (2019). Bruton Tyrosine Kinase Inhibitors: Present and Future. Cancer J. 25 (6), 386–393. 10.1097/PPO.0000000000000412 31764119 PMC7083517

[B13] Cang S. Mukhi N. Wang K. Liu D. (2012). Novel CD20 Monoclonal Antibodies for Lymphoma Therapy. J. Hematol. Oncol. 5, 64. 10.1186/1756-8722-5-64 23057966 PMC3479003

[B14] Chatterjee D. Fatah M. Akdis D. Spears D. A. Koopmann T. T. Mittal K. (2018). "An Autoantibody Identifies Arrhythmogenic Right Ventricular Cardiomyopathy and Participates in its pathogenesis.". Eur. Heart JX 39 (44), 3932–3944. 10.1093/eurheartj/ehy567 PMC6247665 30239670

[B15] Chimen M. McGettrick H. M. Apta B. Kuravi S. J. Yates C. M. Kennedy A. (2015). Homeostatic Regulation of T Cell Trafficking by a B Cell-Derived Peptide Is Impaired in Autoimmune and Chronic Inflammatory Disease. Nat. Med. 21 (5), 467–475. 10.1038/nm.3842 25894827 PMC4425550

[B16] Cordero-Reyes A. M. Youker K. A. Trevino A. R. Celis R. Hamilton D. J. Flores-Arredondo J. H. (2016). Full Expression of Cardiomyopathy Is Partly Dependent on B-Cells: A Pathway that Involves Cytokine Activation, Immunoglobulin Deposition, and Activation of Apoptosis.". J. Am. Heart Assoc. 5. 10.1161/JAHA.115.002484 PMC4859365 26769625

[B17] Curigliano G. Cardinale D. Dent S. Criscitiello C. Aseyev O. Lenihan D. (2016). Cardiotoxicity of Anticancer Treatments: Epidemiology, Detection, and Management. CA Cancer J. Clin. 66 (4), 309–325. 10.3322/caac.21341 26919165

[B18] Douna H. Kuiper J. (2016). Novel B-Cell Subsets in Atherosclerosis. Curr. Opin. Lipidol. 27 (5), 493–498. 10.1097/MOL.0000000000000335 27472410

[B20] Fox E. Lovett-Racke A. E. Gormley M. Liu Y. Petracca M. Cocozza S. (2021). A Phase 2 Multicenter Study of Ublituximab, a Novel Glycoengineered Anti-CD20 Monoclonal Antibody, in Patients with Relapsing Forms of Multiple Sclerosis. Mult. Scler. 27 (3), 420–429. 10.1177/1352458520918375 32351164 PMC7897779

[B21] Frangogiannis N. G. (2014). "The Inflammatory Response in Myocardial Injury, Repair, and remodelling.". Nat. Rev. Cardiol. 11 (5), 255–265. 10.1038/nrcardio.2014.28 24663091 PMC4407144

[B22] García-Rivas G. Castillo E. C. Gonzalez-Gil A. M. Maravillas-Montero J. L. Brunck M. Torres-Quintanilla A. (2020). The Role of B Cells in Heart Failure and Implications for Future Immunomodulatory Treatment Strategies. ESC Heart Fail. 7 (4), 1387–1399. 10.1002/ehf2.12744 32533765 PMC7373901

[B23] Grantomo J. Pratita J. Rachmat J. Saraswati M. (2018). A Rare Case of Primary Cardiac Lymphoma and the Role of Early Surgical Debulking: a Case report.". Eur. Heart J. Case Rep. 2, yty116. 10.1093/ehjcr/yty116 31020192 PMC6425998

[B24] Groschel C. Sasse A. Monecke S. Rohrborn C. Elsner L. Didie M. (2018). "CD8(+)-T Cells with Specificity for a Model Antigen in Cardiomyocytes Can Become Activated after Transverse Aortic Constriction but Do Not Accelerate Progression to Heart Failure.". Front. Immunol. 9, 2665. 10.3389/fimmu.2018.02665 30498501 PMC6249381

[B25] Guo Y. Cen Z. Wei B. Wu W. Zhou Q. (2015). Increased Circulating Interleukin 10-secreting B Cells in Patients with Dilated Cardiomyopathy. Int. J. Clin. Exp. Pathol. 8 (7), 8107–8114. 26339378 PMC4555706

[B26] Haudek S. B. Trial J. Xia Y. Gupta D. Pilling D. Entman M. L. (2008). Fc Receptor Engagement Mediates Differentiation of Cardiac Fibroblast Precursor Cells. Proc. Natl. Acad. Sci. U S A. 105 (29), 10179–10184. 10.1073/pnas.0804910105 18632582 PMC2465805

[B27] Hauser S. L. Waubant E. Arnold D. L. Vollmer T. Antel J. Fox R. J. (2008). B-cell Depletion with Rituximab in Relapsing-Remitting Multiple Sclerosis. N. Engl. J. Med. 358 (7), 676–688. 10.1056/NEJMoa0706383 18272891

[B28] Horckmans M. Bianchini M. Santovito D. Megens R. T. A. Springael J. Y. Negri I. (2018). Pericardial Adipose Tissue Regulates Granulopoiesis, Fibrosis, and Cardiac Function after Myocardial Infarction. Circulation 137 (9), 948–960. 10.1161/CIRCULATIONAHA.117.028833 29167227

[B29] Howlader N. Ries L. A. Mariotto A. B. Reichman M. E. Ruhl J. Cronin K. A. (2010). Improved Estimates of Cancer-specific Survival Rates from Population-Based Data. J. Natl. Cancer Inst. 102 (20), 1584–1598. 10.1093/jnci/djq366 20937991 PMC2957430

[B30] Inaba A. Tuong Z. K. Riding A. M. Mathews R. J. Martin J. L. Saeb-Parsy K. (2020). B Lymphocyte-Derived CCL7 Augments Neutrophil and Monocyte Recruitment, Exacerbating Acute Kidney Injury. J. Immunol. 205 (5), 1376–1384. 10.4049/jimmunol.2000454 32737150 PMC7444279

[B31] Jiao J. Lu Y. Z. Xia N. Wang Y. Q. Tang T. T. Nie S. F. (2018). Defective Circulating Regulatory B Cells in Patients with Dilated Cardiomyopathy. Cell Physiol Biochem 46 (1), 23–35. 10.1159/000488405 29566367

[B32] Keppner L. Heinrichs M. Rieckmann M. Demengeot J. Frantz S. Hofmann U. (2018). Antibodies Aggravate the Development of Ischemic Heart Failure. Am. J. Physiol. Heart Circ. Physiol. 315 (5), H1358–H1367. 10.1152/ajpheart.00144.2018 30095974

[B33] Li Y. Zhou Z. Xin F. Zhang C. Zhang R. Sun D. (2019). Primary Cardiac Lymphoma in Both Atria: A Case Report. J. Clin. Ultrasound 47 (9), 561–563. 10.1002/jcu.22738 31141190

[B34] Lobenwein D. Kocher F. Dobner S. Gollmann-Tepekoylu C. Holfeld J. (2021). "Cardiotoxic Mechanisms of Cancer Immunotherapy - A Systematic review.". Int. J. Cardiol. 323, 179–187. 10.1016/j.ijcard.2020.08.033 32800915

[B35] Ma X. L. Lin Q. Y. Wang L. Xie X. Zhang Y. L. Li H. H. (2019). Rituximab Prevents and Reverses Cardiac Remodeling by Depressing B Cell Function in Mice. Biomed. Pharmacother. 114, 108804. 10.1016/j.biopha.2019.108804 30909146

[B36] Matsui S. Fu M. L. Katsuda S. Hayase M. Yamaguchi N. Teraoka K. (1997). Peptides Derived from Cardiovascular G-Protein-Coupled Receptors Induce Morphological Cardiomyopathic Changes in Immunized Rabbits. J. Mol. Cel Cardiol 29 (2), 641–655. 10.1006/jmcc.1996.0307 9140822

[B37] Matsushita T. Yanaba K. Bouaziz J. D. Fujimoto M. Tedder T. F. (2008). Regulatory B Cells Inhibit EAE Initiation in Mice while Other B Cells Promote Disease Progression. J. Clin. Invest. 118 (10), 3420–3430. 10.1172/JCI36030 18802481 PMC2542851

[B38] Melchers F. (2015). Checkpoints that Control B Cell Development. J. Clin. Invest. 125 (6), 2203–2210. 10.1172/JCI78083 25938781 PMC4497745

[B39] Montecino-Rodriguez E. Dorshkind K. (2012). B-1 B Cell Development in the Fetus and Adult. Immunity 36 (1), 13–21. 10.1016/j.immuni.2011.11.017 22284417 PMC3269035

[B40] Naradikian M. S. Hao Y. Cancro M. P. (2016). Age-associated B Cells: Key Mediators of Both Protective and Autoreactive Humoral Responses. Immunol. Rev. 269 (1), 118–129. 10.1111/imr.12380 26683149

[B41] Nesargikar P. N. Spiller B. Chavez R. (2012). The Complement System: History, Pathways, cascade and Inhibitors. Eur. J. Microbiol. Immunol. (Bp) 2 (2), 103–111. 10.1556/EuJMI.2.2012.2.2 24672678 PMC3956958

[B42] Niederbichler A. D. Hoesel L. M. Westfall M. V. Gao H. Ipaktchi K. R. Sun L. (2006). An Essential Role for Complement C5a in the Pathogenesis of Septic Cardiac Dysfunction. J. Exp. Med. 203 (1), 53–61. 10.1084/jem.20051207 16380509 PMC2118072

[B43] Noutsias M. Pauschinger M. Schultheiss H. U K. (2002). "Phenotypic Characterization of Infiltrates in Dilated Cardiomyopathy - Diagnostic Significance of T-Lymphocytes and Macrophages in Inflammatory cardiomyopathy.". Med. Sci. Monit. 8, CR478–87. 12118194

[B44] Okazaki T. Tanaka Y. Nishio R. Mitsuiye T. Mizoguchi A. Wang J. (2003). Autoantibodies against Cardiac Troponin I Are Responsible for Dilated Cardiomyopathy in PD-1-Deficient Mice. Nat. Med. 9 (12), 1477–1483. 10.1038/nm955 14595408

[B45] Pardoll D. M. (2012). The Blockade of Immune Checkpoints in Cancer Immunotherapy. Nat. Rev. Cancer 12 (4), 252–264. 10.1038/nrc3239 22437870 PMC4856023

[B46] Qin X. Y. Zhang Y. L. Chi Y. F. Yan B. Zeng X. J. Li H. H. (2018). Angiotensin II Regulates Th1 T Cell Differentiation through Angiotensin II Type 1 Receptor-PKA-Mediated Activation of Proteasome. Cel Physiol Biochem 45 (4), 1366–1376. 10.1159/000487562 29462804

[B47] Ramos G. C. van den Berg A. Nunes-Silva V. Weirather J. Peters L. Burkard M. (2017). Myocardial Aging as a T-Cell-Mediated Phenomenon. Proc. Natl. Acad. Sci. U S A. 114 (12), E2420–E2429. 10.1073/pnas.1621047114 28255084 PMC5373357

[B48] Ridker P. M. Everett B. M. Thuren T. MacFadyen J. G. Chang W. H. Ballantyne C. (2017a). Antiinflammatory Therapy with Canakinumab for Atherosclerotic Disease. N. Engl. J. Med. 377 (12), 1119–1131. 10.1056/NEJMoa1707914 28845751

[B49] Ridker P. M. MacFadyen J. G. Thuren T. Everett B. M. Libby P. Glynn R. J., (2017b). Effect of Interleukin-1β Inhibition with Canakinumab on Incident Lung Cancer in Patients with Atherosclerosis: Exploratory Results from a Randomised, Double-Blind, Placebo-Controlled Trial. Lancet 390 (10105), 1833–1842. 10.1016/S0140-6736(17)32247-X 28855077

[B51] Sabatino J. J. Jr. Zamvil S. S. Hauser S. L. (2019). "B-Cell Therapies in Multiple Sclerosis.". Cold Spring Harb Perspect. Med. 9. 10.1101/cshperspect.a032037 PMC6360868 29358322

[B52] Sanchez-Trujillo L. Jerjes-Sanchez C. Rodriguez D. Panneflek J. Ortiz-Ledesma C. Garcia-Rivas G. (2019). Phase II Clinical Trial Testing the Safety of a Humanised Monoclonal Antibody Anti-CD20 in Patients with Heart Failure with Reduced Ejection Fraction, ICFEr-RITU2: Study protocol.". BMJ Open 9, e022826. 10.1136/bmjopen-2018-022826 PMC6475246 30918029

[B53] Savage H. P. Baumgarth N. (2015). Characteristics of Natural Antibody-Secreting Cells. Ann. N. Y Acad. Sci. 1362, 132–142. 10.1111/nyas.12799 26104151 PMC4679694

[B54] Serreze D. V. Fleming S. A. Chapman H. D. Richard S. D. Leiter E. H. Tisch R. M. (1998). B Lymphocytes Are Critical Antigen-Presenting Cells for the Initiation of T Cell-Mediated Autoimmune Diabetes in Nonobese Diabetic Mice. J. Immunol. 161 (8), 3912–3918. 9780157

[B55] Shen P. Fillatreau S. (2015). Antibody-independent Functions of B Cells: a Focus on Cytokines. Nat. Rev. Immunol. 15 (7), 441–451. 10.1038/nri3857 26065586

[B56] Siegel R. DeSantis C. Virgo K. Stein K. Mariotto A. Smith T. (2012). Cancer Treatment and Survivorship Statistics, 2012. CA Cancer J. Clin. 62 (4), 220–241. 10.3322/caac.21149 22700443

[B57] Staudt A. Eichler P. Trimpert C. Felix S. B. Greinacher A. (2007). Fc(gamma) Receptors IIa on Cardiomyocytes and Their Potential Functional Relevance in Dilated Cardiomyopathy. J. Am. Coll. Cardiol. 49 (16), 1684–1692. 10.1016/j.jacc.2006.11.051 17448369

[B58] Thiagaraj A. Kalamkar P. Rahman R. Farah V. Poornima I. (2018). An Unprecedented Case Report of Primary Cardiac Lymphoma Exclusive to Left Ventricle: a Diagnostic and Therapeutic challenge.". Eur. Heart J. Case Rep. 2, yty029. 10.1093/ehjcr/yty029 31020112 PMC6177073

[B59] Torre-Amione G. Kapadia S. Lee J. Durand J. B. Bies R. D. Young J. B. (1996). Tumor Necrosis Factor-Alpha and Tumor Necrosis Factor Receptors in the Failing Human Heart. Circulation 93 (4), 704–711. 10.1161/01.cir.93.4.704 8640999

[B60] Uchida J. Hamaguchi Y. Oliver J. A. Ravetch J. V. Poe J. C. Haas K. M. (2004). The Innate Mononuclear Phagocyte Network Depletes B Lymphocytes through Fc Receptor-dependent Mechanisms during Anti-CD20 Antibody Immunotherapy. J. Exp. Med. 199 (12), 1659–1669. 10.1084/jem.20040119 15210744 PMC2212805

[B61] van den Hoogen P. de Jager S. C. A. Huibers M. M. H. Schoneveld A. H. Puspitasari Y. M. Valstar G. B. (2019). Increased Circulating IgG Levels, Myocardial Immune Cells and IgG Deposits Support a Role for an Immune Response in Pre- and End-Stage Heart Failure. J. Cel Mol Med 23 (11), 7505–7516. 10.1111/jcmm.14619 PMC6815814 31557411

[B62] Waldman A. D. Fritz J. M. Lenardo M. J. (2020). A Guide to Cancer Immunotherapy: from T Cell Basic Science to Clinical Practice. Nat. Rev. Immunol. 20 (11), 651–668. 10.1038/s41577-020-0306-5 32433532 PMC7238960

[B63] Waldmann T. A. (2003). Immunotherapy: Past, Present and Future. Nat. Med. 9 (3), 269–277. 10.1038/nm0303-269 12612576

[B64] Wang L. Li Y. L. Zhang C. C. Cui W. Wang X. Xia Y. (2014). Inhibition of Toll-like Receptor 2 Reduces Cardiac Fibrosis by Attenuating Macrophage-Mediated Inflammation. Cardiovasc. Res. 101 (3), 383–392. 10.1093/cvr/cvt258 24259498

[B65] Wang W. Cai D. (2020). Complement Components sC5b-9 and CH50 Predict Prognosis in Heart Failure Patients Combined with Hypertension. Am. J. Hypertens. 33 (1), 53–60. 10.1093/ajh/hpz140 31429866

[B66] Wu L. Zhao F. Dai M. Li H. Chen C. Nie J. (2017). P2y12 Receptor Promotes Pressure Overload-Induced Cardiac Remodeling via Platelet-Driven Inflammation in Mice. Hypertension 70 (4), 759–769. 10.1161/HYPERTENSIONAHA.117.09262 28827474

[B67] Wu Y. Li Y. Zhang C. Wang X. A. Li H. Du J. (2014). S100a8/a9 Released by CD11b+Gr1+ Neutrophils Activates Cardiac Fibroblasts to Initiate Angiotensin II-Induced Cardiac Inflammation and Injury. Hypertension 63 (6), 1241–1250. 10.1161/HYPERTENSIONAHA.113.02843 24711518

[B68] Yan X. Anzai A. Katsumata Y. Matsuhashi T. Ito K. Endo J. (2013). Temporal Dynamics of Cardiac Immune Cell Accumulation Following Acute Myocardial Infarction. J. Mol. Cel Cardiol 62, 24–35. 10.1016/j.yjmcc.2013.04.023 23644221

[B69] Yanaba K. Hamaguchi Y. Venturi G. M. Steeber D. A. St Clair E. W. Tedder T. F. (2007). B Cell Depletion Delays Collagen-Induced Arthritis in Mice: Arthritis Induction Requires Synergy between Humoral and Cell-Mediated Immunity. J. Immunol. 179 (2), 1369–1380. 10.4049/jimmunol.179.2.1369 17617630

[B70] Youker K. A. Assad-Kottner C. Cordero-Reyes A. M. Trevino A. R. Flores-Arredondo J. H. Barrios R. (2014). High Proportion of Patients with End-Stage Heart Failure Regardless of Aetiology Demonstrates Anti-cardiac Antibody Deposition in Failing Myocardium: Humoral Activation, a Potential Contributor of Disease Progression. Eur. Heart J. 35 (16), 1061–1068. 10.1093/eurheartj/eht506 24375073

[B71] Yu M. Wen S. Wang M. Liang W. Li H. H. Long Q. (2013). TNF-α-secreting B Cells Contribute to Myocardial Fibrosis in Dilated Cardiomyopathy. J. Clin. Immunol. 33 (5), 1002–1008. 10.1007/s10875-013-9889-y 23558825

[B72] Zamorano J. L. Lancellotti P. Rodriguez Muñoz D. Aboyans V. Asteggiano R. Galderisi M. (2016). 2016 ESC Position Paper on Cancer Treatments and Cardiovascular Toxicity Developed under the Auspices of the ESC Committee for Practice Guidelines: The Task Force for Cancer Treatments and Cardiovascular Toxicity of the European Society of Cardiology (ESC). Eur. Heart J. 37 (36), 2768–2801. 10.1093/eurheartj/ehw211 27567406

[B73] Zhang C. Li Y. Wang C. Wu Y. Du J. (2014). Antagonist of C5aR Prevents Cardiac Remodeling in Angiotensin II-Induced Hypertension. Am. J. Hypertens. 27 (6), 857–864. 10.1093/ajh/hpt274 24419904

[B74] Zhang M. Austen W. G. Jr. Chiu I. Alicot E. M. Hung R. Ma M. (2004). Identification of a Specific Self-Reactive IgM Antibody that Initiates Intestinal Ischemia/reperfusion Injury. Proc. Natl. Acad. Sci. U S A. 101 (11), 3886–3891. 10.1073/pnas.0400347101 14999103 PMC374339

[B75] Zhang M. Michael L. H. Grosjean S. A. Kelly R. A. Carroll M. C. Entman M. L. (2006). The Role of Natural IgM in Myocardial Ischemia-Reperfusion Injury. J. Mol. Cel Cardiol 41 (1), 62–67. 10.1016/j.yjmcc.2006.02.006 16781728

[B76] Zouggari Y. Ait-Oufella H. Bonnin P. Simon T. Sage A. P. Guérin C. (2013). B Lymphocytes Trigger Monocyte Mobilization and Impair Heart Function after Acute Myocardial Infarction. Nat. Med. 19 (10), 1273–1280. 10.1038/nm.3284 24037091 PMC4042928

[B77] Zwaka T. P. Manolov D. Ozdemir C. Marx N. Kaya Z. Kochs M. (2002). Complement and Dilated Cardiomyopathy: a Role of Sublytic Terminal Complement Complex-Induced Tumor Necrosis Factor-Alpha Synthesis in Cardiac Myocytes. Am. J. Pathol. 161 (2), 449–457. 10.1016/s0002-9440(10)64201-0 12163370 PMC1850743

